# Salicylic Acid

**Published:** 1910-11

**Authors:** 


### Salicylic Acid.

Salicylic acid, especially its sodium salt, has long been recognized as such a reliable cure for acute rheumatism as to be considered a specific.

From time immemorial the bark of salix alba or willow has been used in intermittent fever and even the Hottentots of South Africa have long used an infusion of some species of willow for the cure of rheumatism.

It is interesting to observe, therefore, just how salicylic acid acts in rheumatism.

There can be no doubt of the fact that rheumatism is caused by some specific micro-organism and that in various articular affections the microbes, when they obtain access to the body, find a suitable nidus in synovial membranes and we know that besides the acute forms, other hybrid forms of pseudo-rheumatisms can be distinguished which are also due to organisms gaining entrance into the joints, such as gonococcus, pneumococcus, staphylococcus, etc.

In addition to the pronounced antiseptic action of salicylic acid it possesses antithermic, analgesic, chologogic and eliminative properties. Besides there are few drugs in the pharmacopeia which can excel sodium salicylate from the natural oil in its action on the liver, for it stimulates this organ to increased activity, causing a greater flow of the bile, which is rendered more watery and is at the same time excreted under a higher pressure.

But one should always use the salicylic acid obtained from natural sources, since this acid has the great advantage of not being a depressant and gives much better results, because it does not contain any of the impurities of the artificial product.

Natural salicylic acid is the remedy par excellence in rheumatism, and, in fact, the micro-organism of acute rheumatism is just as sensitive to natural salicylic acid as that of syphilis to mercury or that of malaria to quinine.

Although not as a specific, as in rheumatism, but as an antiseptic and analgesic, the use of salicylic acid in many other forms of local inflammation, such as tonsilitis, urethritis, orchitis, etc., gives satisfactory results, relieving both pain and distress.

Tongaline not only possesses all the therapeutic properties of salicylic acid from the natural oil, but on account of its other ingredients has a much wider field of usefulness, and is unequaled as an efficient and reliable agent in the treatment of rheumatism, neuralgia, grippe, gout, nervous headache, sciatica, lumbago, malaria, tonsillitis, heavy colds and excess of uric acid.—*Chicago Medical Recorder.*

